# Molecular mechanisms of postintensive care syndrome

**DOI:** 10.1186/s40635-021-00423-6

**Published:** 2021-12-03

**Authors:** Paula Martín-Vicente, Cecilia López-Martínez, Inés Lopez-Alonso, Josefina López-Aguilar, Guillermo M. Albaiceta, Laura Amado-Rodríguez

**Affiliations:** 1 Instituto de Investigación Sanitaria del Principado de Asturias, Oviedo, Spain; 2 Centro de Investigación Biomédica en Red (CIBER)-Enfermedades Respiratorias, Madrid, Spain; 3 Instituto Universitario de Oncología del Principado de Asturias, Universidad de Oviedo, Oviedo, Spain; 4 Critical Care Center, Hospital Universitari Parc Taulí, Institut d’Investigació I Innovació Parc Taulí I3PT, Sabadell, Spain; 5 Unidad de Cuidados Intensivos Cardiológicos, Hospital Universitario Central de Asturias, Oviedo, Spain

Effectiveness of intensive care must be evaluated not only by short-term survival after a critical illness, but also by the recovery to an adequate quality of life. The increased evidence of long-term functional disabilities in intensive care survivors led to the definition of post-intensive care syndrome (PICS) [1]. Early diagnosis and effective treatments for these newly recognized conditions are warranted. However, most of the initial efforts to limit long-term sequelae have not yielded satisfactory results [2]. The objective of this review is to identify the molecular mechanisms that lead to organ dysfunction after intensive care and to summarize them into several plausible unifying hypotheses. From these mechanisms, novel therapeutic targets with the potential to prevent PICS may arise [3], allowing for earlier interventions during acute organ failure aimed to improve the quality of life of intensive care unit (ICU) survivors. Cost-effective strategies based on growing pathogenetic evidence on PICS would hence allocate research efforts and funding to implement preventive treatments, to impede pathogenetic mechanisms triggered during ICU stay, rather than exclusively rehabilitate long-term sequelae when they are already established.

## The spectrum of postintensive care syndrome

The improvement of mortality rates in the ICU has evidenced that survivors to a critical illness face a number of long-term severe complications and sequelae that can impair their quality of life [4]. Several factors, including the population aging along with the emergence of invasive therapies that may improve the outcomes, have increased the interest in these long-term conditions [5]. An expert panel in 2012 defined PICS as the “new or worsening impairments in physical, cognitive or mental health status arising after critical illness and persisting beyond acute care hospitalization” [1]. It must be noted that this definition provides a framework to improve awareness, research and diagnostic and therapeutic approaches, rather to define a classical syndrome [6].

**Fig. 1:**
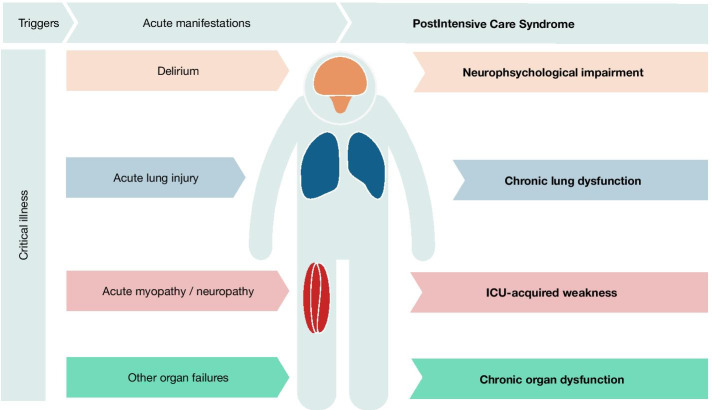
Spectrum of postintensive care syndrome. Many of the long-term conditions that constitute the postintensive care syndrome have been related to specific syndromes that appear during acute care. However, it is not known if this relationship reflects a common primary cause, a pathogenetic association or simply an association due to underlying confounders

PICS covers several dimensions, including physical, cognitive and emotional aspects (Fig. 1), for many of which there is no standard definition or diagnostic criteria. Long-term respiratory sequelae include impairments in lung volumes, ventilatory dynamics and diffusion [7]. Although some studies report a mild impairment in most of the cases, the recent COVID-19 pandemic has highlighted the relevance of the long-term, post-acute respiratory distress syndrome (ARDS) respiratory sequelae [8]. Musculoskeletal impairments are included under the concept of “ICU-acquired weakness” (ICUAW), defined as a “diffuse, symmetric, generalized muscle weakness, detected by physical examination and meeting specific strength related criteria) that develops after the onset of critical illness without other identifiable cause”. ICUAW may result in a severe limitation of daily activities and a significant worsening in quality of life. Although some improvements may occur during the first year after ICU discharge, weakness is persistent in a significant proportion of cases [9]. Finally, neuropsychological alterations regarding cognitive declines in ICU survivors have been also described by several authors. Up to 80% of critically ill patients experience delirium while in the ICU, and a significant number of ICU survivors show signs of moderate cognitive impairment or other neurological, emotional and mental health conditions related to PICS include depression, anxiety, post-traumatic stress disorder and cognitive impairment [10, 11]. Again, these disarrangements may persist well above the first year and cause a severe limitation of patients’ activities.

Several ICU-related risk factors and short-term complications have been related to these long-term outcomes (highlighted in Fig. 1). However, as pathogenetic factors are mostly unknown, it is not clear if these short- and long-term symptoms are time-dependent manifestations of a common disease, independent diseases with shared etiologies or independent sequelae caused by the systemic response to a severe injury.

## Mechanisms of chronic lung dysfunction

**Fig. 2:**
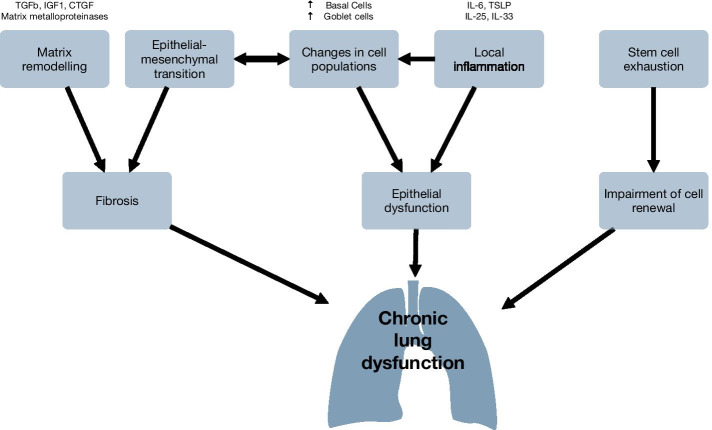
Mechanisms of chronic lung dysfunction after critical illness. Local inflammation, changes in cell populations and matrix remodeling promote a pro-fibrotic state that may cause long-term respiratory impairment. *TRPV4* transient receptor potential cation channel, subfamily V, member 4. *BBB* blood–brain barrier, *DRD2* dopamine receptor type 2

The need of respiratory support is one of the main reasons for ICU admission, either due to lung injury or ventilatory failure. These critically ill patients often require mechanical ventilation. Previous organ damage, along with the use of ventilation, may lead to the development or worsening of lung injury [12], which involves epithelial barrier dysfunction, inflammation and matrix remodelling. In this context, failure to correctly resolve these processes might be involved in the development of long-term sequelae. However, the specific mechanisms by which acute lung damage becomes chronic are yet to be fully elucidated. Most of the knowledge on this topic comes from research on prevalent chronic lung diseases, such as idiopathic pulmonary fibrosis, which may hint to underlying pathogenetic mechanisms. These same processes may play a role in the development of chronic lung dysfunction in the context of post-ICU sequelae (Fig. 2).

Inflammation and matrix remodelling are well described processes involved in the resolution of acute lung injury [13]. However, their perpetuation can be a relevant pathogenetic mechanism of many chronic lung diseases [14]. Persistence of the local inflammatory response has been linked to the development of fibrosis, as released Th2 cytokines (IL-4, -5, -13) have a well-known pro-fibrotic effect and may recruit fibrocytes from the systemic circulation. Moreover, other pro-inflammatory mediators such as IL1b or IL-6 may promote collagen deposition mediated by IL-17 [15].

In this setting, alveolar macrophages, inflammatory cells and fibroblasts release profibrotic molecules during acute injury, such as transforming growth factor-β (TGFβ), Insulin-like growth factor (IGF-1), platelet derived growth factor (PDGF) or connective tissue growth factor (CTGF) among others [16–19]. TGFβ activates intracellular SMAD complexes via binding to serine/threonine kinase heterodimers in the cell surface. Activated SMAD complexes enter the nucleus and act as transcription factors regulating a wide range of cellular processes. In fibrosis, these pathways include extracellular matrix deposition and fibroblast division and differentiation into myofibroblasts [20]. These cells, characterized by an increase in the intracellular content of α-smooth muscle actin (α-SMA), modify the matrix composition by increasing deposition of collagen and disorganizing elastin, generating scar-like lesions [21].

Activation of coagulation and fibrin deposition is another pathogenetic mechanism activated during acute lung injury that has been linked to long-term sequels. It has been shown that patients with lung interstitial diseases have an increased expression of procoagulant factors within the lung, including tissue factor or thrombin, or a decrease in protein C. Activation of proteinase-activated receptors (PARs) by the proteinases from the coagulation cascade (thrombin, trypsin, cathepsins…) provides the mechanistic link between coagulation and fibrosis. Downstream signaling following PAR1 activation results in the expression of several profibrotic growth factors (CTFG, PDGF) and perpetuates the local release of proinflammatory cytokines and TGFβ [22].

Epithelial–Mesenchymal Transition (EMT) has also gained relevance in this scenario, where massive tissue remodeling takes place. EMT is a process in which a polarized epithelial cell acquires a mesenchymal phenotype, that includes synthesis of extracellular matrix components [23]. During EMT, epithelial cells lose part of the epithelial characteristics, such as expression of E-cadherin and cytokeratin, and gain mesenchymal markers including N-cadherin, vimentin or α-smooth muscle actin [24]. At a physiological level, all these processes favour the accumulation of excessive fibrous tissue, decreasing lung compliance and impairing ventilatory dynamics and diffusion. In vivo models of lung injury and mechanical ventilation have shown the activation of EMT, possibly by a *Wnt*-dependent mechanism [25], suggesting a relationship between EMT-like processes and the later development of pulmonary fibrosis [26].

Another common feature in these chronic conditions is epithelial barrier dysfunction, characterized by altered cell composition of the pseudostratified respiratory epithelium with basal and goblet cell hyperplasia and metaplasia [27, 28]. In addition, after the initial lung insult, persistence of epithelial dysfunction is associated with a proinflammatory secretory phenotype due to the activation of airway epithelial cells, dendritic cells and type 2 Innate Lymphoid Cells, and release of epithelial derived cytokines, including thymic stromal lymphopoietin (TSLP), interleukin (IL)-25, and IL- 33 [29]. The resulting sustained inflammation and shift in cellular composition could play an important role in post-ICU lung dysfunction.

Finally, cell renewal is a key feature of chronic lung diseases. Excessive stem cell activation leads to accumulation of DNA damage and cell senescence [30]. In patients with idiopathic pulmonary fibrosis, epithelial cells show an increase expression of senescence markers, such as P16 or P21 and a proinflammatory phenotype [31]. Recently, we have shown the activation of this pathway in response to acute lung injury [32]. These senescent cells have been related to stem cell exhaustion with an impaired regenerative capacity [33] and an increased secretion of inflammatory and matrix remodeling molecules, which in turn may perpetuate fibrosis.

## Molecular mechanisms of cognitive impairment

There are several mechanisms that may lead to brain injury in critically ill patients [34]. The central nervous system (CNS) receives signals from neural afferences and circulating factors and cells. Regarding the former, the vagus nerve constitutes the main ascendent pathway from peripheral organs. Distal vagal sensors are responsive to a variety of stimuli, including stretch (via transient receptor potential cation channel, subfamily V, member 4 [TRPV4] and Piezo receptors) [35], or inflammation (via toll-like receptor (TLR)-4, IL1R or tumor necrosis factor [TNF]-receptor present in vagal sensory neurons) [36, 37]. Once these signals reach the brain stem, multisynaptic pathways along the CNS are activated [38, 39]. For instance, lung stretch activates alveolar TRPV4 and purinergic receptors, that, in a vagus-dependent manner, increase dopaminergic signaling and triggers hippocampal apoptosis [40]. Blockade of triggering receptors in distal organs or circulating mediators could decrease the risk of long-term impairment. In animal models, inhibition of peripheral mechanosensation with TRPV4 antagonists, unspecific blockade of nerve conduction with lidocaine or inhibition of type 2 dopamine receptors have decreased hippocampal apoptosis [35].

Circulating molecules and cells may also reach the brain during critical illness. The systemic inflammatory response decreases blood–brain barrier permeability [41] and facilitates the translocation of circulating mediators and/or cells that further promote brain injury. Heparan sulfate fragments released from the endothelial glicocalix during sepsis may translocate to the hippocampus and inhibit brain-derived neurotrophic factor signaling, that results in memory impairment in mice [42]. Circulating IL-6 may also play a role in this setting, as peripheral blockade of IL-6 with a monoclonal antibody prevented ventilator-induced brain injury [43]. In line with these findings, intratracheal instillation of lipopolysaccharide increases the expression of proinflammatory cytokines *Il1b* and *Il6* in the brain stem [44]. Interestingly, only the increase in *Il1b* expression was abolished after vagotomy, suggesting the simultaneous activation of different mechanisms.

**Fig. 3:**
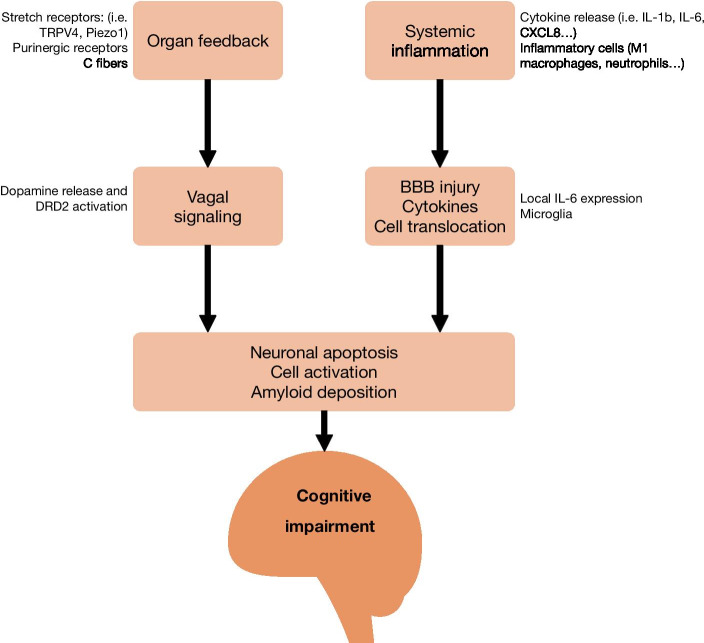
Mechanisms of brain damage after critical illness. Neural and circulating signals reach the brain during critical illness and trigger several pathogenetic responses, including neuronal apoptosis, cell activation and amyloid deposition, that could explain the long-term sequelae experienced by ICU survivors

The link between these brain responses and functional outcomes has also been assessed. In a large animal model of prolonged protective mechanical ventilation, hippocampal damage was demonstrated [45]. Acute lung damage and mechanical ventilation in mice caused brain inflammation, hippocampal injury and memory impairments, in an steroid-preventable manner [41]. Similarly, conditioning responses, a surrogate marker of memory in mice, were absent 3 days after mechanical ventilation, but not in anesthetized, non-ventilated controls [46]. Although translation of these experimental results into clinical evidence is challenging and remains elusive, this model of brain injury in response to systemic insults (summarized in Fig. 3) provides a framework for prevention, diagnosis and treatment of long-term cognitive dysfunction in critically ill patients.

## Mechanisms of ICU-acquired weakness

ICUAW is a bilateral and symmetrical neuromuscular involvement, common in critically ill mechanically ventilated patients. Clinical studies in critical care settings involving electrophysiological tests and muscle histopathology suggest that both polyneuropathy and myopathy may coexist in ICU patients, being myopathy more frequently identified as the cause of weakness [47]. Critical illness neuropathy has been described as a distal axonal sensory-motor polyneuropathy affecting limb and respiratory muscles. Some evidence suggests that weakness recovery could be worsened and/or delayed when neuropathy accompanies myopathy, being persisting disability associated with polyneuropathy and myopathy coexistence [48, 49]. Because nerve conduction studies and needle electromyography do not accurately discern between both entities, and given the sufficiently relevant clinical problem of muscle weakness in these patients [50], the term ICUAW emerged regardless of its causative nature. Although physical disability related to ICUAW is highly prevalent among ICU survivors, its clinical spectrum varies not only in severity but also in recovery trajectories [51]. Muscle atrophy in the critically-ill has been demonstrated to begin within the first hours after ICU admission in mechanically ventilated patients [52] and its development has been related to several factors, such as systemic inflammation, severity of the underlying disease, use of neuromuscular blockers or mechanical ventilation itself [49, 53, 54].

**Fig. 4:**
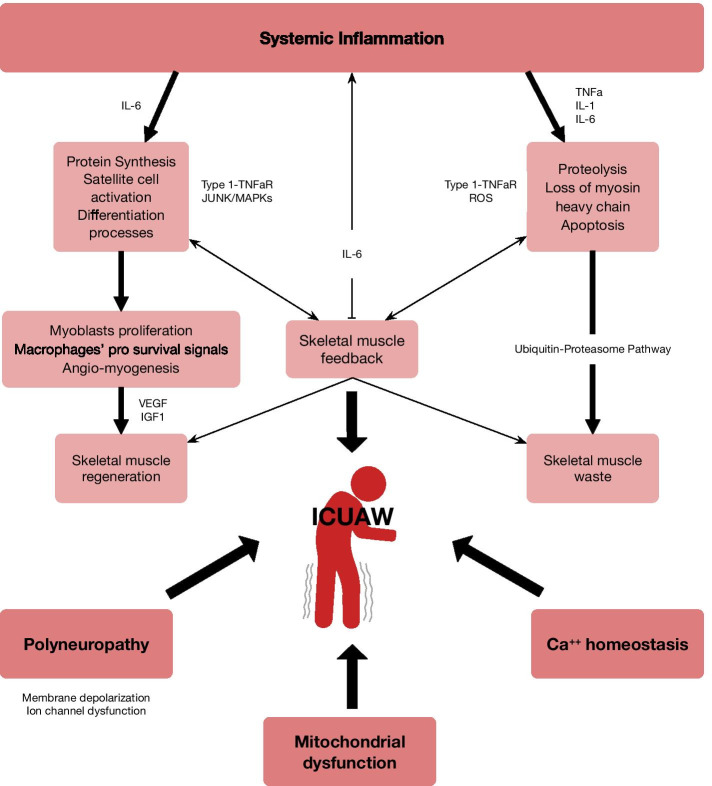
Mechanisms of ICU-acquired weakness. Systemic inflammation, polyneuropathy and local impairment of mitochondria and calcium influx result in a disbalance between protein synthesis and degradation, leading to muscle dysfunction

Multiple molecular mechanisms, either independent or interacting, are involved in muscle wasting and evolve over time, from the onset of critical illness till the long-term recovery phase around 6 months after ICU discharge [55] (Fig. 4). Muscle wasting results from an increased proteolysis triggered in the acute phase, overwhelming the regenerative capacity of the injured tissue [52, 56]. Early activation of proteolytic pathways, however, is not sustained over time, but instead it may alter muscle biology resulting in an impaired muscle regrowth [57].

Inflammatory cytokines have been suggested to play a relevant role in the development of ICUAW. TNFα has been widely studied in this setting. In differentiated myotubes, TNFα stimulates catabolism by binding to TNF receptor subtype 1 and activating nuclear factor-kB. This transcriptional factor is essential for TNFα-induced reduction in muscle protein and loss of adult myosin heavy chain content [58], which is specifically found in critically ill patients [59]. This pathway is also sensitive to reactive oxygen species, which appear to function as second messengers for TNFα in skeletal muscle [58]. TNFα/nuclear factor-kB signaling is also involved in the differentiation process, representing a mechanism that could be responsible for satellite cells activation and skeletal muscle recovery following the acute phase [60–62]. TNFα binding to its receptor also stimulates apoptosis and Jun-N-terminal kinases and mitogen-activated protein kinases (MAPKs) in differentiated myotubes. In muscle cells, these signaling events stimulate the expression of genes related to the ubiquitin–proteasome pathway [63, 64], triggering massive intracellular proteolysis [65]. Finally, TNFα is also known to affect the force of muscle contraction even in the absence of atrophy [66] via TNFR1 and mediated by an increased cytosolic oxidant activity [67, 68].

Elevated levels of IL-1 are commonly found in critically ill patients’ serum and represent a potential stimulus for protein loss and muscle atrophy. The suggested underlying mechanisms are related to both protein synthesis and degradation [69, 70]. Interestingly, IL-6 has been proven to drive the systemic compensatory anti-inflammatory response syndrome, by inhibiting TNFα release and stimulating IL-10 [71]. In skeletal muscle, IL-6 is involved in myogenesis, lipid metabolism, glucose uptake and both protein synthesis and degradation [72–74]. Skeletal muscle cellular niche has been recognized itself as a myokine secretor organ and even a potential regulator of immune system [75]. In mechanically ventilated patients who developed myopathy, the inflammation-induced acute phase response resulted in a marked increase in IL-6 production in skeletal muscle [76].

Histologic and molecular analyses performed in skeletal muscle biopsies of critically ill patients suggest that recovery failure may be associated with satellite/progenitor cells loss and fibrosis [57], but it is unclear which are the underlying mechanisms leading to the satellite cell depletion or what is the role of the whole skeletal muscle cell niche. In other scenarios, where muscle injury may occur, muscle tissue repair is a complex biological process that necessarily involves activation of stem cells. Myogenic stem cells, so-called satellite cells, reside beneath the basal lamina of muscle fibers [77] and express both NCAM/CD56 and early myogenic cell markers, such as M-cadherin, PAX7, and MYF5 [78]. Satellite cells remain quiescent in skeletal muscle, but they can proliferate and further differentiate into myoblasts in response to activating signals, achieving muscle regeneration [79]. Activated satellite cells may interact with macrophages recruited at the site of muscle regeneration and receive mitogenic signals from these immune cells, mediated by the release of different soluble factors [80]. Myoblasts, and to a higher extent myotubes, also receive cell-contact-mediated pro-survival signals from macrophages [81].

Similarly, the microvascular niche seems to be another partner of satellite cells. Christov et al. have suggested quiescent satellite cells to easily interplay with endothelial cells upon activation to set up coordinated angio-myogenesis in a functional manner [82]. Indeed, angiogenesis and myogenesis could share regulatory factors such as vascular endothelial growth factor (VEGF) [83] or insulin-like growth factor type 1 (IGF1) [84] that reciprocally signal both processes pivotally involved in muscle regeneration.

Apart from inflammation and stem cell depletion, several other mechanisms may be involved in the development of persistent muscle weakness in survivors of critical illness. Distal axonal sensory-motor damage/dysfunction has also been described in ICUAW, being attributable to a reduced membrane excitability resulting from membrane depolarization and ion channel dysfunction [85, 86], together with an altered calcium homeostasis [87]. In critically-ill patients with demonstrated polyneuropathy, motor axons are depolarized. Chronic membrane depolarization could be related to tissue hypoperfusion or to increased extracellular potassium in patients with kidney failure, and may lead to muscle atrophy [88]. Reduced compound motor action potentials are present in neuropathy and myopathy. In addition, there have been described fibrillation potentials or positive sharp waves that could be explained by denervation or by muscle sodium channel dysfunction [89]. Mitochondrial dysfunction could be a contributing defect involved in energy-dependent processes [90], and a dysregulation in autophagy [91] has also been described to play a role in muscle repair.

In critically ill patients, the limited sample size of the muscle specimens precludes the identification of different mechanism-specific subphenotypes of muscle weakness, with different histopathological findings and driven by different mechanisms, though leading to the wide clinical spectrum of long-term functional disability. These different pathogenetic subphenotypes could explain the heterogeneous recovery observed among survivors of critical illness. For instance, the activated pathways could be promoting proliferation of satellite cells in some patients while related to fibrotic repair and satellite cell depletion in others. Understanding these mechanisms is crucial to identify therapeutic targets that, interfered at the beginning of the process, could modify the clinical course of ICUAW before the 6-month plateau has been achieved, either at the acute phase or during recovery.

## Unifying hypotheses

Long-term sequelae included in the PICS framework may be the consequence of organ-specific mechanisms, as described in the previous sections. However, the variety of symptoms included in PICS in response to common triggers (the critical disease and its managements) and the observed links and correlations amongst the different dimensions of the syndrome [92] raise the hypothesis that PICS is the long-term result of underlying mechanisms activated by severe diseases, that become systemic and lead to different degrees of multi-organ dysfunction. There are several stereotypical biological responses and mechanisms that could be involved in the development of PICS. Identification of these shared mechanisms could help to identify patients at risk of developing sequelae. Moreover, this knowledge could lead to novel therapeutic interventions that might prevent the whole spectrum of PICS by interfering with upstream regulators.

### Systemic inflammation

The most characterized and studied mechanism in this setting is the inflammatory response. Inflammation is a physiological response necessary to fight against infections or injury and, therefore, restore homeostasis. During the acute phase of the inflammatory response, the presence of damage or pathogen-associated molecular patterns (DAMPs and PAMPs respectively) induces an initial systemic inflammatory response, mediated by the release of pro-inflammatory mediators, such as growth factors (i.e., G/GM-CSF, FltL) and cytokines (i.e., IL-1, IL-6, IL-17) as well as through mesenchymal or immune cells [93]. This coexists with a compensatory anti-inflammatory response syndrome, which is mainly carried out by myeloid suppressor cells (MDSCs) that secrete anti-inflammatory cytokines (e.g., IL-10 and TGFβ) and cytokine antagonists (e.g., IL-1ra and sTNFRI) and decrease inflammation without eliminating all protective innate immunity [94].

During chronic inflammation, an unbalance between pro- and anti-inflammatory mediators makes homeostasis impossible to restore. The persistence of stimuli that modify the inflammatory response, either directly or indirectly, may perpetuate the release of inflammatory mediators not only to the lung but also to the systemic compartment, as the increased alveolo-capillary permeability facilitates their translocation. This persistent inflammation has been demonstrated in ARDS survivors even after clinical improvement or recover and related to worse physical recovery [95].

### Senescence

Other potential mechanism which might be involved in PICS is related to the activation and spread of senescent responses. Cell senescence is defined as the cell cycle arrest in response to a stimulus, and a shift towards a specific phenotype characterized by the loss of several cell functions and the paracrine release of a variety of molecules (termed senescence-associated secretory phenotype—SASP), including proinflammatory mediators and senescence inductors (thus activating a positive feedback) [96]. There are several molecular pathways that lead to senescence in response to injury (including oxidative stress, release of DAMPs, proinflammatory cytokines, such as TNFα or IL-1α), most of which depend on the activation of P53, P21 and their downstream factors [97]. In a model of acute lung injury, we demonstrated that activation of these pathways results in short-term protection against apoptosis (as senescent cells have an inherent resistance to programmed cell death) [32, 98]. However, the resulting senescent state could lead to long-term sequelae, both locally and in distant organs (in response to SASP). Several of the previously described mechanisms of organ-specific PICS could be manifestation of these senescent responses. Senescence is an emerging pathogenetic pathway in idiopathic lung fibrosis [31], but their involvement in secondary fibrosis is unknown. Several forms of acute lung injury can trigger senescence in response to DNA damage. The previously described satellite cell exhaustion in ICUAW could be also a manifestation of the systemic release of pro-senescent factors. Recently, it has been described the deposition of brain amyloid fibers, a known trigger of neuronal senescence, in response to critical illness [99].

Of note, there is a positive feedback between inflammation and senescence. Inflammation causes activation of senescence through several mediators, such as IL-6. In turn, SASP includes the release of proinflammatory molecules, thus promoting a sustained response [100].

### Integrated stress response

A third mechanism potentially involved in the development of PICS is the so-called integrated stress response (ISR). ISR is a conserved cell response to different pathological conditions that results in a general decrease in protein synthesis and expression of a specific gene signature. Its activation is necessary to maintain cell homeostasis in the presence of different stress signals [101]. This response may be activated by four stress-sensing kinases (protein kinase R [PKR], Eukaryotic translation initiation factor 2-alpha kinase 4 [EIF2AK4], heme-regulated inhibitor [HRI] and PKR-like endoplasmic reticulum kinase [PERK]) that phosphorylate the eukaryotic initiation factor elF2α, which ultimately leads to a decrease in protein synthesis and the induction of selected genes (such as ATF4 and CHOP) that eventually take part in the cellular response to stress.

ISR plays an important role during acute lung injury or mechanical ventilation. Alveolar overdistension induced by mechanical ventilation results in PERK phosphorylation and subsequent phosphorylation of the factor elF2α. This alters epithelial permeability, induces proinflammatory cytokine release and cell death [102]. ISR may have a dual role deciding cell fate. Although its main function is maintaining cell survival, exposure to a continuous stress could lead to cell death [103]. In this context, the stress-inducible phosphatase GADD34 dephosphorylates elF2α and induces a negative feedback mechanism, ceasing the activation of ISR [104]. However, in pathological conditions, ISR is activated but GADD34 expression is attenuated, thus preserving phosphorylated elF2α [105] and perpetuating this response [106] due to the lack of negative feedback.

### From common triggers to cellular and tissue dysfunction

Finally, all these pathways converge in a reduced number of cell responses that mediate tissue dysfunction. The most studied response is apoptosis. There is substantial evidence showing disseminated apoptosis during the acute response to critical illness [107]. This programmed cell death is activated by binding of extracellular signaling molecules (i.e., TNFα) to membrane receptors or intracellular release of cytochrome c from injured mitochondria (i.e., after oxidative stress). These pathways converge in the activation of caspases, that lead to DNA fragmentation and cell death. Although apoptosis is a major pathogenetic mechanism in acute organ failure, and anti-apoptotic drugs may prevent organ damage in this setting, the relationship of acute apoptosis and development of PICS remains to be fully elucidated. Animal models have shown a correlation between apoptosis and later development of pulmonary fibrosis [108] and neurological deficits [46], but human data is scarce. Patients with ICUAW show activation of proapoptotic pathways in peripheral muscles [109].

Activation of senescence, that render cells resistant to apoptosis [110], may be a compensatory mechanism in this setting, but at the price of the promoting persistence of senescent, dysfunctional cells. Senolytics, a heterogeneous family of compounds that inhibit pro-survival kinases in senescent cells (such as *BCL2* or tyrosine-kinases), may promote the selective death of these cells, facilitating a delayed repair [111]. Although promising, these pathways have not been explored in critically ill patients.

## Emerging PICS domains

Rather than a settled syndromic condition, PICS is an evolving concept that covers a large variety of symptoms and conditions experienced by ICU survivors. Clinical research in this topic may help to better identify, characterize and manage the long-term consequences of critical illness from the early onset of acute illnesses. The main components of PICS have been covered in the previous sections, but the systemic nature of acute severe diseases may cause other organ injuries.

Critical illness may increase the risk of cardiovascular events after ICU and hospital discharge. Greater rates of atrial fibrillation, heart failure, and myocardial infarction have been described after sepsis [112]. However, no clear organ-specific mechanisms have been identified to date [113]. Systemic persistent inflammation is a major driver of cardiovascular disease, but a clear causative link is missing [114]. Recently, the role of stress-triggered senescence has been highlighted in this setting [115].

Similarly, there is increasing evidence of long-term impaired kidney function after critical care, even in patients without acute renal failure [116]. The development of kidney fibrosis during the repair phase has been proposed as a major pathogenetic mechanism [117]. In addition, cell cycle arrest, a hallmark of senescence, promotes fibrosis during kidney repair [118]. The previously described cardiovascular impairment could contribute to further deteriorate kidney function.

Finally, transgenerational effects of critical illness, requiring modification of the genome or epigenome of germline cells, have barely been explored. It has been shown that experimental sepsis changes the sperm methylome, mainly in intergenic regions or development-related genes [119]. Both systemic and local inflammation can modify the expression of methyltransferases and thus facilitate cell reprogramming. The consequences of these changes in offspring, however, are controversial [120]. Besides, maternal prenatal exposures in human studies have focused on pregnancy, rarely assessing long term effects of exposures of maternal non-pregnant progenitor in later offspring [121].

## Conclusions

The objectives of intensive care must go well beyond ICU survival and aim to provide critically ill patients with the best achievable quality of life. This includes prevention, treatment and/or palliation of the long-term sequels derived from their ICU stay. The complex organ crosstalk and the pleiotropic effects of most of the responses triggered by critical illness make difficult to find treatments that translate into a clinical benefit. In this difficult scenario, knowledge of the underlying biological mechanisms may allow clinicians and researchers to identify novel biomarkers, therapeutic targets and strategies that ultimately will facilitate the identification and treatment of these long-term sequelae even at early and acute stages, thus contributing to improve long-term outcomes.

## Data Availability

Not applicable.
